# Internet of Things (IoT) Operating Systems Management: Opportunities, Challenges, and Solution

**DOI:** 10.3390/s19081793

**Published:** 2019-04-15

**Authors:** Yousaf Bin Zikria, Sung Won Kim, Oliver Hahm, Muhammad Khalil Afzal, Mohammed Y. Aalsalem

**Affiliations:** 1 Department of Information and Communication Engineering, Yeungnam University, 280 Daehak-Ro, Gyeongsan, Gyeongbuk 38541, Korea; yousafbinzikria@gmail.com; 2 Zühlke Group, Eschborn, 65760 Hessen, Germany; oliver.hahm@zuehlke.com; 3 Department of Computer Science, COMSATS University Islamabad, Wah Campus, Wah Cantt 47010, Pakistan; khalilafzal@ciitwah.edu.pk; 4 Department of Computer Networks, Jazan University, Jazan 45142, Saudi Arabia; aalsalem.m@gmail.com

**Keywords:** IoT, IoT OS, IIoT, WSN, UWSN, ICN, Smart Home, Smart City, VANETS, SDN, Edge Computing

## Abstract

Internet of Things (IoT) is rapidly growing and contributing drastically to improve the quality of life. Immense technological innovations and growth is a key factor in IoT advancements. Readily available low cost IoT hardware is essential for continuous adaptation of IoT. Advancements in IoT Operating System (OS) to support these newly developed IoT hardware along with the recent standards and techniques for all the communication layers are the way forward. The variety of IoT OS availability demands to support interoperability that requires to follow standard set of rules for development and protocol functionalities to support heterogeneous deployment scenarios. IoT requires to be intelligent to self-adapt according to the network conditions. In this paper, we present brief overview of different IoT OSs, supported hardware, and future research directions. Therein, we provide overview of the accepted papers in our Special Issue on IoT OS management: opportunities, challenges, and solution. Finally, we conclude the manuscript.

## 1. Introduction

Internet of Things (IoT) is the main driving force behind revolutionizing all aspects of technology. The seamless integration of all the technologies is the challenging task [1]. Recent advancements in Millimeter Wave (mmWave) [2], emerging cellular networks [3], Fifth Generation (5G) spectrum potential for intelligent IoT [4], caching techniques in cellular networks [5], coexitense of wireless technologies [6], fog computing [7], Vehicular to Everything (V2X) [8], Device to Device (D2D) Communications [9], IoT resources [10], and IoT Operating Systems (OS) [11,12] etc. research paves the way towards next generation IoT. Advancements in IoT technologies and availability at lower prices thriving the devices connectivity and remote accessibility. Hence, the adaptation of standards are essential to allow communication among these heterogeneous networks in IoT. Connectivity of industry components using central or distributed manners to increase the productivity and efficiency is must for Industrial IoT (IIoT). The fourth industrial revolution or industry 4.0 is still evolving. It opens up humongous challenges for smart and autonomous system that generates huge amount of data to be processed and hence needs to be intelligent by adopting machine learning algorithms.

IoT OS continuous development by practitioners and researchers are crucial to provide platform that supports latest protocols standard for the future intelligent IoT. The device heterogeneity in IoT is challenging however, IoT OS needs to support different hardware architectures, boards and devices. There are variety of IoT OSs namely Contiki-OS [13], RIOT [14], and Zephyr [15] are available to facilitate tremendous growth in this area. IoT devices are resource-constrained in terms of hardware resources and usually with limited battery capacity. Consequently, well known mature OS cannot be run on these devices. IoT OS code requires to be optimized with essential Transmission Control Protocol/Internet Protocol (TCP/IP) capabilities for seamless integration with the global internet. Therefore, IoT OS needs to be very efficient to manage the resources on all the communication layers.

Majority of IoT OSs provides complete IP networking stack with standard User Datagram Protocol (UDP) [16], TCP [17] and Hypertext Transfer Protocol (HTTP) [18]. Moreover, it also supports latest standards like Internet Protocol version 6 (IPv6) over Low-Power Wireless Personal Area Networks (6LoWPAN) [19], Routing over Low Power and Lossy Networks (ROLL), and Constrained Application Protocol (CoAP) [20].

The rest of the paper is organized as follows. Section 2 briefly discusses the IoT OSs key features and characteristics. Section 3 deliberates the supported hardware. Section 4 provides future research directions. Section 5 summarizes the accepted paper. Finally, Section 6 concludes the paper.

## 2. IoT OS Key Features and Characteristics

Table 1 provides the key features of different IoT OSs. The list of IoT OSs are exhaustive. Therefore, we only considered that is mostly used by the research community. TinyOS [21] is preliminary designed for Wireless Sensor Networks (WSN) and distinctively most popular among the research community for many years. However, nowadays it is not used much by the researchers due to lack of active development. TinyOS uses dialect of C programming language called nesC. This complex customized language is hard to learn. It follows the monolithic architecture and provides cooperative task scheduler. Tinythread [22] can be used to achieve the multi-threading. It also provides IPv6 stack based on 6LoWPAN. TinyOS Low-Power Listening (LPL) implements the Radio Duty Cycling (RDC) to provide the energy efficiency and consequently enhances the network lifetime. TinyOS provides discrete event simulator called TOSSIM. Hence, users can run and debug the program on the system instead of the mote.

Contiki [23] is actively developed by the practitioners and research community. Therefore, it is widely used by the research community for IoT constrained devices. The low memory requirements make Contiki well suited for low power constrained devices. It is written in C language. It also provides the multithreading using the protothread. Contiki uses the cooperative or preemptive based scheduling for the processes. Contiki supports several rich network stacks that provides comprehensive set of features like IPv6, 6LoWPAN, RPL and CoAP. Moreover, it also provides multiple industry standard Medium Access Control (MAC) such as Carrier Sense Multiple Access (CSMA) and Time Slotted Channel Hopping (TSCH). ContikiMAC and Contiki X-MAC RDC is used to make the motes energy efficient. Cooja simulator or emulator is used to quickly write, test and debug the code before actual deployment. It supports numerous IoT devices like wismote, sky and, z1. Cooja is written in java and implemented as a single simulation thread. Hence, it cannot take advantage of multi-core processors and takes long time to finish up the simulation for dense network scenarios. Further, it needs to further develop to accommodate newly available IoT hardware platforms.

RIOT [24] is developed on top of microkernel named FireKernel by team of freie University Berlin and HAW Hamburg. Ever Since, active developer and research community is growing and adding the desired industry specific standards to support ongoing research. The design goals include energy efficiency, small memory footprint, modularity and uniform API access that provides independent hardware abstraction. RIOT supports C and C++ programming languages. It also provides multithreading with tickless, preemptive and priority based scheduler. Multithreading is designed to reduced inherent drawbacks such as thread management overhead, code stack usage, and inter-process messaging. Native is the emulator or hardware virtualizer that allows the user to run the RIOT code as a linux processes. Hence, it is easier to develop IoT software without the need of actual hardware.

Zephyr is originally developed by Intel subsidiary wind river. It provides microkernel for less constrained IoT devices and nanokernel for constrained devices.It supports multithreading with cooperative, priority-based, Earliest Deadline First (EDF), non-preemptive and preemptive scheduling. The programs can be written in C and C++ programming language. Zephyr provides network stack support with multiple protocols. It also support Bluetooth Low Energy (BLE) 5.0. The applications can be develop, build and test using the native posix port.

MbedOS [25] the Real Time Operating System (RTOS) is developed by Advanced RISC Machine (ARM) for constrained IoT devices. It is specifically designed for 32 bit ARM architecture. It is based on monolithic kernel and provides preemptive scheduler. It supports C and C++ development. MbedOS features multithreading, 6LoWPAN, BLE, WiFi, sub-GHz, Near Field Communication (NFC), Radio-Frequency Identification (RFID) and Long Range Low-Power Wide Area Network (LoRaLPWAN). Low memory requirements and various hardware support of mbedOS makes it suitable for IoT research and development.

Formerly brillo and now the android things [26] is developed by Google. It is based on android however, it is simplified and trimmed down android version to run on low-power IoT devices. It supports development in both C and C++ programming language. It is built on top of monolithic kernel and provides completely fair scheduler. Android things memory requirements makes it unsuitable for low-end constrained IoT devices rather it is designed for high-end IoT devices.

## 3. IoT OS Supported Motes

IoT OS support of widely used IoT constrained devices are crucial. Table 2 lists the board architecture build by different vendors that are supported by IoT OSs. Most of these devices have the small to medium-level resources. The small IoT resource constraint devices usually contains 10 KB of Random Access Memory (RAM) and 100 KB of Read Only Memory (ROM). Whereas, medium IoT resource constraint devices have more than of 10 KB of RAM and 100 KB of ROM. Thus, it allows richer applications with advance protocols and secure communication. Except android things rest of the IoT OS is well suited for low to medium constrained IoT devices. Small IoT devices are specialized devices and pose a strict requirement on IoT OS to be very hardware specific with limited capabilities. Medium IoT devices provides a flexibility to include complete IP suite and different applications to run on top of network stack. Further, the devices provide additional functionalities and can act as internet router, host or a server.

## 4. Future Research Directions

***Energy efficiency*** is the crucial aspect for the IoT. Majority of the IoT devices are resource constrained in nature. Therefore, battery or other constrained energy sources are used to operate it. IoT deployment scenarios are diverse, challenging and sometimes in very remote areas. Humongous IoT network size demands IoT OS to be energy efficient to run the IoT devices for many years. IoT employs RDC to achieve the energy efficiency. Efficient techniques are required to achieve the accurate motes synchronization along with the RDC.

***Real time capabilities*** of the IoT motes is crucial for timely execution of critical tasks. Internet of Body (IoB) requires to meet hard deadlines to achieve certain task. Real time operating system (RTOS) is specifically designed to guarantee completion of these tasks within the certain time frame. Therefore, IoT OS should have the capability to act as RTOS as well.

***Network connectivity*** is essential for upward and download traffic. Multi-interface may be used to provide multi-homing or to communicate on different spectrum frequencies. Continuous evolution and availability of heterogeneous industry standard protocols at different layers is desirable to provide seamless integration and connectivity of motes to form networks.

***Security and safety*** of critical systems such as health care, smart home, smart city etc. are highly desirable. In general, IoT OS should support security and privacy of overall IoT network. Open challenges include data integrity, authentication and access mechanisms. Blockchain based optimized solution is one of the promising viable technique to address the privacy and security in the IoT. Further, the deployed network solutions should be continuously reviewed to fix the bugs. Quick development, deployment, testing and adaptation to recent proposed security standards are essential to provide the ultimate network security.

***Small memory footprint*** of IoT OS with the availability of complete TCP/IP stack to run on highly constrained devices is crucial to integrate seamlessly with the global internet. The optimization of the modules in memory efficient manner without loosing any functionality is a trivial task. To achieve this, designers and developers have to follow the coding conventions with high degree of ease of configurability and modularity is desirable.

***Heterogeneous devices support*** is ncessary for the IoT OS. The rapid growth of IoT with diverse use cases leads the way to develop massive heterogeneous devices. Hence, IoT OS needs to constantly incorporate newly developed hardware platform. Explosion of numerous IoT OSs and heterogeneous devices pose another challenge called interoperability. Thus, there should be standard set of rules for development of multiple layers of protocol by the IoT OSs. Consequently, deployment scenarios with multiple use cases that contains heterogeneous devices, running different IoT OSs can seamlessly integrate without an issue.

***Intelligent IoT*** (I-IoT) are the future key contributor for massive adaptation of IoT in daily life. In recent years, researchers start exploring and applying the artificial intelligence (AI) to IoT use cases. Machine learning (ML) is well explored and investigated in the area of neural networks and image processing. However, its potential and application is not yet fully explored for IoT. ML techniques needs to be optimized to run on IoT constrained devices.

***IoT and big data*** is closely linked together as IoT devices generate humongous amount of unstructured data. Therefore storage, processing and analyzing big data is essential to generate the meaningful reports to make decisions. This can lead to data-driven research instead of hypothesis driven. New efficient and accurate techniques of data analytics is crucial for development of future innovative solutions.

## 5. A Brief Review of Articles of This Special Issue

Proliferation of IoT devices leads to the dense wireless network deployments. Further, availability of sub-1 GHz bands for the communication requires standard to fully explore the potential. Thus, IEEE 802.11ah [27] standard is released. In 802.11ah, a single Service Access Point (SAP) can support and serve maximum of 8191 stations with a minimum of 100 kbps data rate within the range of 1 km. In [28], authors explored Cognitive Radio (CR) based 802.11ah networks and proposed new distributed MAC protocol named as carrier sense Restricted Access with Collision and Interference Resolution (RACIR). Its the hybrid MAC protocol based on CSMA/CD and CSMA/CR protocols. It resolves the scalability and hidden primary terminal problem. Each station enable contention free grouping access by estimating the active stations in the group by using split algorithm.Therein, it access the channel in random access contention based manner to avoid interference with the Primary User (PU) receiver. RACIR is compared with CR-CSMA/CA and the results showed that it considerably improves performance in terms of throughput, delay and energy consumption.

IIoT is revolutionizing the industry. It is used to collect, analyze and apply the information from numerous sensors to further improve the overall efficiency of the system. The network design and topology plays a crucial role on the performance of the system. The proper handling of massive data generated by the sensors and security are the key to run the system efficiently. Therefore, Liu et al. [29] addressed the topology design by considering uncertain factors in production process and sensor demands. Moreover, they proposed big data analysis model along with the security protection system. They used Analytic Hierarchy Process (AHP) to analyze the intelligent evaluation index model. They applied and evaluated the proposed solution to the diesel engine enterprise. According to the results, the proposed network topology deployment interconnect all the production units, efficiently process, handle and share the information among different units, and increased the system security. Consequently, it makes the enterprise more robust, adaptive and flexible.

Smart homes with efficient Home Energy Management System (HEMS) provides reliability and consequently energy conservation. These systems are specifically designed to cater challenges like user comfort, and cost reduction etc. In [30] Ain et al. presented Fuzzy Inference System (FIS) by considering humidity level as an additional parameter. They also used indoor room temperature variation as a feedback to FIS to manage the energy consumption and user comfort. They also automate the FIS using automatic rule based generation method using the combinatorial method. The results showed that the proposed scheme improves the overall system and considerably reduced the energy consumption without compromising the user comfort.

Underwater Wireless Sensor Networks (UWSNs) is useful for aquatic monitoring, pollution monitoring and mineral extraction etc. Highly challenging UWSNs communication environment makes it very difficult to route the packets from sensor nodes to the sink. These devices are mostly battery operated and highly resource constrained. Hence, inefficient routing method consumes more energy and inevitably results in node failures. These sudden node failures results in creating void node problem. Sher et al. in [31] proposed four schemes namely Adaptive transmission range in WDFAD-Depth-Based Routing (DBR) (A-DBR), Cluster-based WDFAD-DBR (C-DBR), Backward transmission-based WDFAD-DBR (B-DBR) and Collision Avoidance-based WDFAD-DBR (CA-DBR) to increase energy efficiency, decrease void node problem, decrease end-to-end delay, fall back recovery mechanism and reduce collisions. A-DBR dynamically adjust the transmission range to cater void node problem and consequently save energy with higher successful packet delivery. C-DBR decreases end-to-end delay but on the cost of higher energy consumption. B-DBR increases packet delivery ratio with increase in overall accumulative propagation distance. CA-DBR consumes less energy along with low end-to-end delay. Hence, different proposed schemes improves the quality in certain aspects and provides performance trade-offs according to user requirement.

Information-Centric Networking (ICN) uses name instead of IP address to retrieve the contents. ICN faces many challenges in emerging and dynamic environment such as Vehicular Ad Hoc Networks (VANETS). Din et al. [32] discussed the comprehensive opportunities and challenges for ICN with respect to Software Defined Network (SDN), cloud computing and edge computing. They discussed aforementioned models challenges and future research directions in terms of mobility, security, routing, naming, caching and 5G communications.

Implementation of edge computing is a trivial task for constrained IoT devices. IoT OS manages all the resources of IoT motes. In [33] Rodriguez-Zurrunero et al. investigated thoroughly the cross-influence of computational load of different processing tasks for IoT devices. Communication and processing are two inter-related tasks. Authors used YetiOS [34] that is built on top of FreeRTOS and YetiMotes for testbed. Certain communications scheme have strict timing requirements to complete the task. Otherwise, overall system performance degrades significantly. It is very crucial for healthcare or real time surveillance to process the information on time and generate the alarms or alerts accordingly. Hence, availability of affordable additional computational resources is necessary for handling high load and faster communication.Contrarily, design of intelligent communication protocol is required to handle the high communication load. Hence, new process management schemes can manage the communication tasks and processing tasks efficiently and fulfill the requirements of both. Additional experiments with different IoT OSs by considering transport protocols, routing protocols, MAC protocols, and complex deployment scenarios to study the other aspects is crucial for better understanding of cross-effects between processing and communication tasks.

Blockchain is proposed to act as a shared and decentralized ledger to keep the transaction records.There are three main types of blockchain namely public, private and consortium. Public blockchains are decentralized management systems and allows anonymous participants. Private blockchain is for single organization where only trusted and identified users are permitted to participate. Consortium blockchains are designed for multiple organizations with trusted and identified participants. Public blockchain technologies are very slow as compared to private or consortium blockchains technologies. Obour Agyekum et al. [35] proposed secure and efficient re-encryption blockchain scheme for resource constrained IoT network. Experiment results revealed that the proposed scheme increases the processing delay however at the cost of secure interactions between the entities. Further studies are required to reduce processing delay and make it more efficient.

CoAP is a specialized protocol specifically designed for low cost constrained IoT networks. Fully fledged CoAP requires extensive computing, processing and storage capabilities. Therefore, to cope with this issue, Islam et al. [36] proposed CoAP handler for ICN POINT architecture. The objective is to provide CoAP group communication without IP multicast support and changing existing Domain Name System (DNS). Moreover, they added the functionality of CoAP observe and enable delaying response when CoAP server is in sleep mode. Experiments are conducted on POINT testbed and in mininet. Results shows that proposed scheme successfully able to shift the overhead and complexity from the CoAP endpoints to the ICN network without loosing any functionality. CoAP observe aggregation scheme also reduces the communication overhead. Further evaluation on larger testbed is required to fully see the potential of proposed scheme.

Khalid et al. [37] proposed spatial and temporal spectral-hole sensing framework for Full Duplex enabled Secondary User (FD-SU) Transmitters (TXs) deployed in IoT CRN(IoT-CRN) spectrum heterogeneous environment. They incorporated the proposed sensing model and present the analytical formulation. They evaluated the Utilization of Spectrum (UoS) scheme for FD-SU TXs present at different spatial positions.It is demonstrated that self-interference, primary user (PU) activity level, and the sensing outcomes in spatial and temporal domains have a significant influence on the utilization performance of spectrum. The FD-SUTX in R2 (with spatial opportunity) have the excessive false alarms. However, the average number of successful secondary communicating sensing slots for FD-SU TX in region one (R1) (with only temporal opportunity) are less than that of FD-SU TX in region two (R2). Owing to the fact that FD-SU TX in R2 can avail the spatial spectral opportunities even when PU is in ON state, which is not the case for FD-SU TX in R1. It is interesting to consider and evaluate the temporal and spatial variations of idle channels in more complicated IoT-CRN scenarios.

IoT based Intelligent Transportation Systems (ITS) are crucial for road safety and are essentially part of the smart cities. Cheaper Smart phone sensors based ITS solutions are rather preferred over expensive hardware based solutions. Bhatti et al. [38] presented ITS solution to reduce the false positive rates. The proposed scheme contains accident detection and notification system. They used accelerometer, Global Positioning System (GPS), pressure and microphone sensors to correctly detect the accidents and informs the medical rescue team for immediate medical assistance. The results shows that proposed system performs better than the past related schemes. However, it is essential to test the proposed scheme in real time scenarios to fully realize the effectiveness of the system before actual deployment.

## 6. Conclusions

Ten papers in this SI presents state-of-the-art research trend in the area of IoT OS management, opportunities, challenges, and solutions. The papers presented interesting discussion and novel ideas for the readers. The guest editors would like to show appreciation to authors and thank all the anonymous reviewers on providing constructive feedback to improve the overall quality of all the accepted papers. We would also like to thank editor-in-chief Prof. Dr. Vittorio M.N. Passaro, Prof. Dr. Leonhard M. Reindl, Prof. Dr. Assefa M. Melesse, Prof. Dr. Alexander Star and managing editor Fanny Fang for the invaluable help and productive advice in finalizing this SI.

## Figures and Tables

**Table 1:** Overview of IoT OSs.

OS	Min RAM	Min ROM	C	C++	Multi	Architecture	Scheduler
			Support	Support	Threading		
TinyOS	<1 kB	<4 kB	✗	✗	∼	Monolithic	Cooperative
Contiki	<2 kB	<30 kB	∼	✗	∼	Monolithic	Cooperative,
							preemptive
RIOT	∼1.5 kB	∼5 kB	✓	✓	✓	Microkernel	Tickless,
							Preemptive,
							Priority based
Zephyr	∼2 kB to ∼8 kB	∼50 kB	✓	✓	✓	Nanokernel,	Preemptive,
						Microkernel	Priority based
MbedOS	∼5 kB	∼15 kB	✓	✓	✓	Monolithic	Preemptive
brillo	∼32 MB	∼128 MB	✓	✓	✓	Monolithic	Completely Fair

Note: ∼ Partial Support; ✓ Support; ✗ No Support.

**Table 2:** IoT OSs Supported Boards.

IoT OS	AVR	MSP430	ARM	x86	ARC	PIC32
TinyOS	✓	✓	✓	✗	✗	✓
Contiki	✓	✓	✓	✗	✓	✓
RIOT	✓	✓	✓	✓	✗	✓
Zephyr	✗	✗	✓	✓	✓	✗
MbedOS	✗	✗	✓	✗	✗	✗
brillo	✗	✗	✓	✓	✗	✗

Note: ✓ Support; ✗ No Support.

## Abbreviations

The following abbreviations are used in this manuscript:

5G	Fifth generation
6LoWPAN	IPv6 over Low-Power Wireless Personal Area Networks
AHP	Analytic Hierarchy Process
AI	Artificial Intelligence
BLE	Bluetooth Low Energy
CoAP	Constrained Application Protocol
CR	Cognitive Radio
CSMA	Carrier Sense Multiple Access
D2D	Device to Device
EDF	Earliest Deadline First
FD-SU	Full Duplex enabled Secondary User
FIS	Fuzzy Inference System
HEMS	Home Energy Management System
HTTP	Hypertext Transfer Protocol
ICN	Information-Centric Networking
IIoT	Industrial IoT
ITS	Intelligent Transportation System
IoT	Internet of Things
I-IoT	Intelligent Internet of Things
IoT-CRN	IoT- Cognitive Radio Network
LoRaLPWAN	Long Range Low-Power Wide Area Network
MAC	Medium Access Control
ML	Machine Learning
mmWave	MillimeterWave
NFC	Near Field Communication
OS	Operating Systems
PU	Primary User
RACIR	Restricted Access with Collision and Interference Resolution
RDC	Radio Duty Cycling
RFID	Radio-Frequency Identification
ROLL	Routing over Low Power and Lossy Networks
RTOS	Real Time Operating System
SAP	Service Access Point
SDN	Software Defined Network
TCP	Transmission Control Protocol
TSCH	Time Slotted Channel Hopping
UDP	User Datagram Protocol
UoS	Utilization of Spectrum
UWSNs	Underwater Wireless Sensor Networks
V2X	Vehicular to Everything
VANETS	Vehicular Ad Hoc Networks
WSN	Wireless Sensor Networks
